# The Influence Mechanism of Extracurricular Activity Participation on the Perceived Improvement of Comprehensive Literacy Among Medical Students: The Mediating Role of Self-Efficacy

**DOI:** 10.7759/cureus.96742

**Published:** 2025-11-13

**Authors:** Wenyang Li, Xinmin Jiang, Minghui Zhang, Yannuo Ma, Chunran Zhao

**Affiliations:** 1 College of Dental Medicine, Qilu Medical University, Zibo, CHN; 2 Department of Business Administration, Joongbu University, Goyang, KOR

**Keywords:** comprehensive literacy, extracurricular activities, mediating effect, medical students, self-efficacy, structural equation modeling

## Abstract

Objectives: The present study aims to examine how participation in extracurricular activities influences medical students’ perceived improvement in comprehensive literacy. Specifically, it seeks to analyze the direct effect of extracurricular activity participation on perceived improvement in comprehensive literacy, investigate the mediating role of self-efficacy in this relationship, and validate a structural equation model illustrating the interrelationships among these variables. In this study, comprehensive literacy was operationalized as students' self-perceived improvement in critical thinking, innovation, collaboration, responsibility, and social concern.

Methods: A questionnaire survey was conducted in July 2025 with 264 valid participants (sophomore to fifth-year students) from Qilu Medical University. The perceived improvement of comprehensive literacy was measured using an adapted scale based on the "4Cs" framework, comprising six items that assessed dimensions such as multi-perspective reflection, creative application, collaboration, taking initiative, and social concern. Measures also included the General Self-Efficacy Scale and self-reported participation frequency. Data were analyzed using IBM Corp. Released 2018. IBM SPSS Statistics for Windows, Version 27.0. Armonk, NY: IBM Corp. for one-way ANOVA and correlation analysis, and AMOS 29.0 for structural equation modeling with a bootstrap procedure (5000 samples) to test the mediating effect.

Results: One-way ANOVA revealed significant differences in comprehensive literacy perceived improvement scores across different participation frequency groups (F=5.436, p<0.001), with a trend of higher scores associated with more frequent participation. Confirmatory factor analysis indicated a good measurement model fit (χ²/df=2.386, CFI=0.994, RMSEA=0.073). Path analysis confirmed that participation frequency not only directly and positively predicted comprehensive literacy perceived improvement (β=0.197, p<0.001) but also had an indirect effect through enhancing general self-efficacy. The mediating effect of self-efficacy was significant (effect=0.073, 95% CI [0.004, 0.106]), accounting for 27.04% of the total effect.

Conclusion: Extracurricular activity participation is a significant pathway for promoting comprehensive literacy development among medical students, with higher participation frequency leading to more pronounced effects. General self-efficacy plays a partial mediating role in this relationship. Universities should encourage students to actively participate in extracurricular activities and intentionally foster their self-efficacy within these activities to more effectively enhance their comprehensive literacy.

## Introduction

The United Nations Educational, Scientific, and Cultural Organization (UNESCO) emphasizes that the core mission of higher education is to cultivate responsible citizens capable of addressing global challenges and promoting sustainable and peaceful development. Similarly, the World Bank points out that in the knowledge economy era, the goal of education is to empower students with comprehensive abilities that foster economic growth, social inclusion, and environmental sustainability. Medical students will face complex public health challenges, rapidly evolving medical technologies, and diverse patient needs, requiring them to possess not only superb professional skills but also profound humanistic literacy, ethical judgment, a global health perspective, and interdisciplinary collaboration capabilities [1]. However, traditional medical education models often focus heavily on knowledge delivery in the first classroom (formal curriculum), while the cultivation of students' comprehensive literacy largely relies on the practice and experience gained through extracurricular activities. Although numerous studies affirm the positive value of extracurricular activities, a fundamental question remains insufficiently answered: How does the frequency of participation in extracurricular activities affect the improvement of medical students' comprehensive literacy? What is the underlying psychological mechanism of action? Delving into this "black box" mechanism is an urgent need for optimizing talent training models and enhancing the quality of talent development.

While the positive value of extracurricular activities is acknowledged in general higher education, their specific role and underlying mechanisms within medical education remain comparatively underexplored. Furthermore, existing research often remains at the level of exploring simple correlations between extracurricular activity participation and student development outcomes, with in-depth analysis of the internal influence paths or mechanisms still lacking. Therefore, a theoretical framework that systematically explains the interaction between environment, individual cognition, and developmental outcomes is needed to reveal the mediating mechanism therein. From the perspective of social cognition, there is a continuous, bidirectional interaction between personal factors, environment, and behavior [2]. Within this framework, general self-efficacy is regarded as a key psychological mechanism connecting the external environment with individual development outcomes [3]. Rao's research indicates that when students overcome challenges and achieve success in extracurricular activities, their belief in their own abilities is enhanced [4]. This enhanced belief is not confined to a specific activity domain but can generalize into a "general self-efficacy," influencing students' performance across multiple areas. Verner-Filion et al. found a positive association between self-determined motivation for extracurricular activities and various student functional indicators [5]. Although this literature primarily focuses on motivation, high self-efficacy is a key factor promoting self-determined motivation. Therefore, general self-efficacy is likely an important bridge connecting extracurricular activity participation and the improvement of comprehensive literacy.

## Materials and methods

Participants

The questionnaire survey for this study was conducted in July 2025, targeting sophomore to fifth-year medical students at Qilu Medical University. Participants were selected using a stratified random sampling method to ensure representativeness across different academic years. Before the formal survey, the research team revised and confirmed the scale wording and number of items through expert interviews and a small-scale pilot test. At the beginning of the questionnaire, an introductory statement clearly informed participants of the purpose of the study, emphasized the voluntary nature of participation, explained that all responses would remain confidential and be used for research purposes only, and included an informed consent statement requiring participants to confirm their understanding before proceeding. A total of 306 questionnaires were collected in the formal survey. After screening out invalid questionnaires, including those with excessively short completion times, consecutive identical responses, and incorrect answers to attention check questions, 264 valid questionnaires were obtained, resulting in a valid response rate of 86.8%. After data entry, IBM Corp. Released 2018. IBM SPSS Statistics for Windows, Version 27.0. Armonk, NY: IBM Corp. and AMOS 29.0 software were used for reliability and validity tests, one-way ANOVA for group comparison, and path model estimation.

Questionnaire tools and variable measurement

The questionnaire for this study consisted of three parts: basic information, the Comprehensive Literacy Perceived Improvement Scale, and the Self-Efficacy Scale. The Comprehensive Literacy Perceived Improvement Scale was adapted from the "4Cs" theoretical framework [6]. It was contextualized to reflect the learning characteristics of university students' extracurricular activities. The scale comprises 6 items and uses a 5-point Likert scale, ranging from 1 ("strongly disagree") to 5 ("strongly agree"). Through exploratory factor analysis and confirmatory factor analysis, this scale demonstrated a single-factor structure in this study with good reliability, and the model fit indices met standards. Specific reliability and validity analyses are presented in the results section.

General self-efficacy was measured using the Chinese version of the General Self-Efficacy Scale, originally developed by Ralf Schwarzer and colleagues and adapted into Chinese by Zhang and Schwarzer [7-9].

The Chinese version consists of 10 items and has been widely used among Chinese university student populations, with previous studies demonstrating its good reliability and validity [10]. In accordance with the scale's usage permissions, the full scale is not reproduced in the Appendix; only one sample item is provided: “I can always manage to solve difficult problems if I try hard enough.” In this study, the Cronbach's α coefficient for this scale was 0.936, indicating excellent internal consistency reliability.

Extracurricular activity participation frequency was measured by participants' self-report of "how many times they participated in school-organized extracurricular activities last semester." It was categorized into five groups based on participation frequency: 0 times (non-participation), 1-3 times, 4-6 times, 7-9 times, and 10 times or more. This variable was included in the analysis as the independent variable.

Research design and hypotheses

Extracurricular Activity Participation and Student Development

Extracurricular activities refer to various organized, purposeful activities that students voluntarily participate in outside the prescribed curriculum, with the core purpose of supplementing and expanding classroom teaching and promoting students' holistic development [11]. These activities cover multiple fields such as academics, arts, sports, community service, and leadership cultivation, aiming to provide students with opportunities for practical experience, skill development, and interpersonal communication. Broadly speaking, extracurricular activities are an indispensable part of the educational process, playing a crucial role especially in cultivating 21st-century core skills like innovation capability, critical thinking, communication, and collaboration.

Substantial research indicates that extracurricular activities significantly enhance various qualities and abilities of university students. For example, the study by Wu and Fernando emphasizes that extracurricular activities can effectively promote the development of students' innovation skills, including creativity, critical thinking, communication, and collaboration, which are essential for students to adapt to social changes and enhance their employability [12]. Hammoda also notes that extracurricular activities are increasingly recognized for cultivating practical skills in entrepreneurial learners, closely linking entrepreneurship courses with real life through social situational learning experiences, stimulating student cognition, and promoting reflective practice [13].

For medical students, the role of extracurricular activities in enhancing comprehensive literacy is equally prominent. Aryal points out that extracurricular activities in the medical field, whether research-related or not, can help medical students develop key skills required to become excellent doctors, including resume building, teamwork, stress management, and effective communication [11]. Atta et al., through optimizing extracurricular activities using an integrative medicine approach, found that this effectively improved student satisfaction, indicating that well-designed extracurricular activities can meet the needs of medical students and promote their holistic development [14]. By participating in various extracurricular activities such as medical societies, research projects, and volunteer services, medical students can acquire practical experience and interpersonal skills difficult to gain in the classroom, enhancing their empathy, professional responsibility, and ability to solve complex problems.

Based on this, this study proposes hypothesis H1: Extracurricular activity participation has a significant positive impact on the perceived improvement of comprehensive literacy among medical students.

The Role of General Self-Efficacy in the Perceived Improvement of Comprehensive Literacy Among Medical Students

General self-efficacy is a core construct of social cognitive theory, referring to an individual's overall belief in their ability to cope with challenges across a wide range of situations [7]. Unlike domain-specific self-efficacy, it reflects a cross-situational level of confidence. Individuals with high self-efficacy exhibit greater resilience in the face of difficulties and tend to set higher goals and exert more effort [15].

In the context of medical education, this belief system is crucial for the professional development and comprehensive literacy enhancement of medical students. Research shows that general self-efficacy is closely related to medical students' academic motivation, self-regulated learning, and learning engagement, collectively predicting their academic achievement [16,17]. Specifically, medical students with high self-efficacy demonstrate better academic performance and more positive emotional experiences [18]; they are also more adept at using deep learning strategies, actively integrating resources, and seeking feedback to optimize their professional performance [19]. Furthermore, self-efficacy has been proven to be a key positive factor in medical education, helping students adapt to the learning environment, and is closely related to their professional persistence and mental health [20].

In summary, as a key personal cognitive resource, general self-efficacy empowers medical students to proactively navigate academic and professional challenges. By positively influencing their learning motivation, strategy application, and adaptive behaviors, it facilitates their holistic development in terms of knowledge acquisition, skill development, and professional identity and integrity. Based on it, this study proposes hypothesis H2: General self-efficacy has a significant positive impact on the perceived improvement of comprehensive literacy among medical students.

The Mediating Role of Self-Efficacy

Social cognitive theory provides the core theoretical framework for understanding the relationship between extracurricular activities and self-efficacy. This theory emphasizes that individual cognition forms and develops through continuous interaction with the environment. Extracurricular activities, as a typical "environmental" factor, contain challenges, practical opportunities, and social interactions that are key sources for building self-efficacy through mastery experiences and social persuasion [7].

Specifically, extracurricular activities provide students with a practical field to apply knowledge and solve problems. The sense of mastery gained from successfully overcoming these challenges is the most effective way to enhance an individual's general confidence. Empirical studies have observed this positive pathway in different student populations. For instance, among general university students, extracurricular activities have been proven to be an important context for developing student innovation capability, a process inevitably accompanied by successful experiences and confidence accumulation [12]. A study in an open and distance learning environment further confirmed that students' general self-efficacy significantly predicts their academic achievement [21], conversely suggesting that any educational intervention that can enhance self-efficacy may positively impact student learning and development.

For medical students, extracurricular activities are explicitly recognized as a core pathway for cultivating key professional competencies such as teamwork and stress management [11]. The acquisition and enhancement of these abilities directly and powerfully reinforce their "I can do it" self-belief [20]. Based on the above theoretical and empirical evidence, this study proposes H3: Extracurricular activity participation frequency has a significant positive impact on medical students' general self-efficacy.

Furthermore, general self-efficacy, as a core personal cognitive resource, is considered a key mediating mechanism connecting environmental stimuli and individual development outcomes. Research generally indicates that general self-efficacy can significantly predict students' academic achievement [21] and plays an important mediating role in the influence of other variables on problem-solving ability [22]. This means that extracurricular activities may enhance medical students' general self-efficacy, thereby increasing their resilience, strategy use, and cognitive engagement when facing academic and professional challenges, ultimately leading to a comprehensive improvement in their comprehensive literacy. Therefore, we infer that general self-efficacy is the internal psychological mechanism explaining how extracurricular activities work. Based on this, this study proposes H4: General self-efficacy plays a mediating role in the relationship between extracurricular activity participation frequency and the perceived improvement of comprehensive literacy.

Research Mode

**Figure 1:**
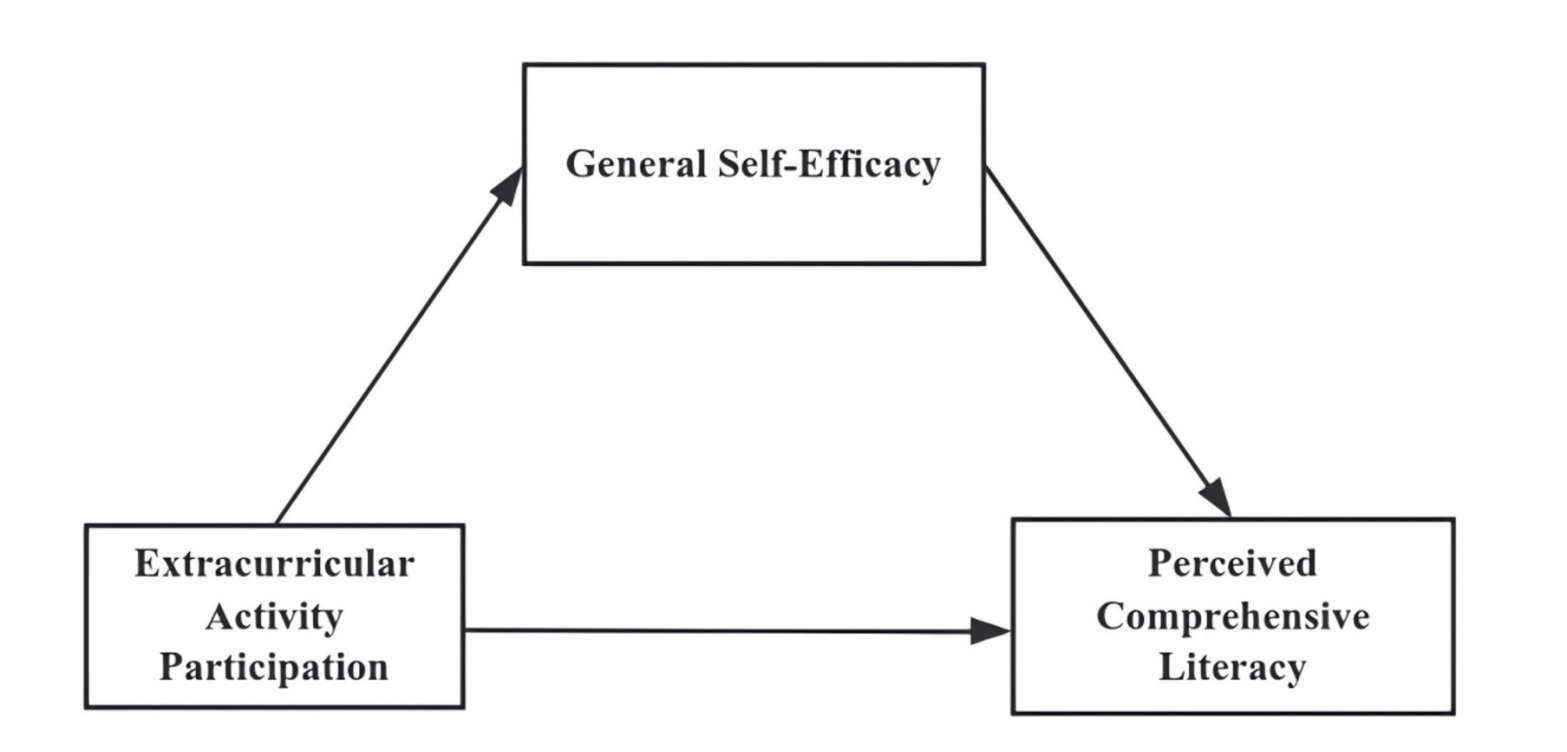
Theoretical hypothesis model

Based on the above literature review and hypothesis development, this study constructed the theoretical model shown in Figure 1. The model hypothesizes that extracurricular activity participation frequency (independent variable) both directly affects the perceived improvement of comprehensive literacy (dependent variable) and produces an indirect effect through general self-efficacy (mediating variable).

Data analysis

A one-way ANOVA was first performed to test for significant between-group differences in comprehensive literacy perceived improvement scores based on extracurricular activity participation frequency. Subsequently, LSD post-hoc comparisons were conducted to pinpoint the specific groups between which these differences existed. This step provided critical distributional insights and a parameter basis for the ensuing path modeling.

Subsequently, reliability and validity tests were conducted on the adapted scales, including exploratory factor analysis and confirmatory factor analysis. IBM Corp. Released 2018. IBM SPSS Statistics for Windows, Version 27.0. Armonk, NY: IBM Corp. was used for descriptive statistics and Pearson correlation analysis. On this basis, AMOS 29.0 software was used to construct a structural equation model. The model analysis steps were as follows: First, assess the overall model fit and the significance of each path coefficient; second, use the bias-corrected bootstrap method (5000 resamples, 95% confidence interval) to estimate the mediating effect. If the 95% confidence interval of the mediating effect did not include 0, the indirect effect was considered statistically significant.

## Results

Sample descriptive analysis

This study collected a total of 264 valid questionnaires. The sample distribution is presented in Table 1. Among the participants, 109 (41.3%) were male and 155 (58.7%) were female, indicating a relatively balanced gender distribution. Regarding academic year, sophomores constituted the largest proportion (n=152, 57.6%), followed by juniors (n=51, 19.3%), seniors (n=37, 14.0%), and fifth-year students (n=24, 9.1%). The higher proportion of lower-grade students might be attributed to their greater compliance and more active participation in questionnaire surveys. Future studies could consider increasing the sample size of upper-grade students to enhance the representativeness and generalizability of findings. In terms of extracurricular activity participation frequency during the past semester, 13 (4.9%) students reported no participation, while 121 (45.8%) participated 1-3 times. Participants engaging in activities 4-6 times, 7-9 times, and ≥10 times accounted for 61 (23.1%), 29 (11.0%), and 40 (15.2%) of the sample, respectively.

**Table 1:** Descriptive statistical analysis of the sample (N=264)

Variable	Category	Count	Percentage %	Cumulative Percentage
Gender	Male	109	41.30%	41.30%
	Female	155	58.70%	100%
Year of Study	Sophomore	152	57.60%	57.60%
	Junior	51	19.30%	76.90%
	Senior	37	14.00%	90.90%
	Fifth-Year	24	9.10%	100%
Frequency of Participation in Extracurricular Activities (Past Semester)	Never	13	4.90%	4.90%
	1-3 times	121	45.80%	50.80%
	4-6 times	61	23.10%	73.90%
	7-9 times	29	11.00%	84.80%
	10times or more	40	15.20%	100%

Variable descriptive and correlation analysis

To explore the preliminary relationships between variables, this study used the Pearson Correlation Coefficient for correlation analysis. Correlation analysis is a statistical method used to measure the strength and direction of the linear relationship between two variables. Its value ranges between -1 and +1. A positive value indicates a positive correlation, a negative value indicates a negative correlation, and values closer to ±1 indicate a stronger correlation. This analysis helps determine whether there are significant relationships between variables, providing a basis for subsequent structural equation modeling and mediation effect testing.

As shown in Table 2, the mean score for extracurricular activity participation frequency was 2.86 (SD = 1.16), for general self-efficacy was 3.06 (SD = 0.57), and for comprehensive literacy perceived improvement was 4.02 (SD = 0.83). The correlation test results showed a significant positive correlation between extracurricular activity participation frequency and general self-efficacy (r = 0.140, p < 0.05) and a significant positive correlation between extracurricular activity participation frequency and comprehensive literacy perceived improvement (r = 0.261, p < 0.01). Meanwhile, the correlation coefficient between general self-efficacy and comprehensive literacy perceived improvement was 0.548 (p < 0.01), both reaching significant levels.

**Table 2:** Variable descriptive statistics and correlation coefficient matrix *p<0.05 **p<0.01 ***p<0.001

	Mean	Standard Deviation	Extracurricular Activity Participation	General Self-Efficacy	Perceived Comprehensive Literacy
Extracurricular Activity Participation	2.86	1.16	1		
General Self-Efficacy	3.06	0.57	0.140^*^	1	
Perceived Comprehensive Literacy	4.02	0.83	0.261^**^	0.548^**^	1

One-way ANOVA

To examine whether there were significant differences in the comprehensive literacy perceived improvement levels among medical students with different frequencies of extracurricular activity participation, a one-way analysis of variance (One-Way ANOVA) was conducted. The extracurricular activity participation frequency was divided into 5 groups (1="never", 2="1-3 times", 3="4-6 times", 4="7-9 times", 5="10 times or more"), with the comprehensive literacy perceived improvement score as the dependent variable.

First, the results of the homogeneity of variance test revealed a Levene statistic of 1.554 and a significance p-value of 0.187 (p > 0.05). This indicates that the variances are homogeneous across groups, satisfying the prerequisite assumption for ANOVA. The results of the descriptive statistical analysis (Table 3) demonstrated that as the frequency of extracurricular activity participation increased, the average score for perceived improvement in comprehensive literacy exhibited a clear upward trend (rising from 3.37 points in the "Never" group to 4.43 points in the "10 times or more" group). This preliminarily suggests a positive association between the two.

**Table 3:** Comparison of comprehensive literacy perceived improvement scores by extracurricular activity participation frequency group (N=264)

Participation Frequency Group	N	Mean	Standard Deviation
Never	13	3.37	1.16
1-3 times	121	3.91	0.81
4-6 times	61	4.04	0.81
7-9 times	29	4.15	0.71
10 times or more	40	4.43	0.68
Total	264	4.02	0.83

The results of the one-way ANOVA (Table 4) indicated that the between-group differences reached a statistically significant level (F(4, 259) = 5.436, p < 0.001). This means that there were significant differences in the comprehensive literacy perceived improvement scores among medical students with different frequencies of extracurricular activity participation.

**Table 4:** One-way ANOVA summary table

Source of Variation	Sum of Squares (SS)	Degrees of Freedom (df)	Mean Square (MS)	F-value	Significance (p)
Between Groups	13.973	4	3.493	5.436	<0 .001
Within Groups	166.422	259	0.643		
Total	180.396	263			

To further clarify the specific differences between the groups, this study used the LSD method for post-hoc multiple comparisons. The results (Table 5) showed: the comprehensive literacy perceived improvement score of the "Never" group (1) was significantly lower than that of the "1-3 times" group (2) (p = 0.022), the "4-6 times" group (3) (p = 0.007), the "7-9 times" group (4) (p = 0.004), and the "10 times or more" group (5) (p < 0.001).

**Table 5:** Summary of significant post-hoc multiple comparisons (LSD method) *p < 0.05, **p < 0.01, ***p < 0.001, this table only lists between-group comparisons that are statistically significant.

(I) Participation Frequency	(J) Participation Frequency	Mean Difference (I-J)	Significance (p)
Never	1-3 times	-0.539	0.022*
	4-6 times	-0.664	0.007**
	7-9 times	-0.778	0.004**
	10 times or more	-1.053	< 0.001***
1-3 times	10 times or more	-0.515	<0 .001***
4-6 times	10 times or more	-0.389	0.018*

The score of the "1-3 times" group (2) was significantly lower than that of the "10 times or more" group (5) (p < 0.001).

The score of the "4-6 times" group (3) was significantly lower than that of the "10 times or more" group (5) (p = 0.018).

The differences between other group pairs did not reach statistical significance (p > 0.05).

The one-way ANOVA results confirmed that the frequency of extracurricular activity participation has a significant impact on medical students' perceived improvement. Specifically, students who had participated in extracurricular activities had significantly higher perceived improvement levels than those who never participated. Moreover, among the participating students, the high-frequency participation group ("10 times or more") had a significantly higher comprehensive literacy perceived improvement level than the low-frequency participation groups ("1-3 times" and "4-6 times"). These findings provide important preliminary evidence for subsequently constructing a structural equation model to further explore the causal relationships between variables.

Reliability test

To examine the internal consistency of the latent variable scales, this study calculated Cronbach's alpha coefficients. The results are shown in Table 6. The "General Self-Efficacy" scale, comprising 10 items, had an overall Cronbach's alpha of 0.936. Deleting any single item did not significantly increase the coefficient, indicating good internal consistency among the items. The "Comprehensive Literacy Perceived Improvement" scale had a Cronbach's alpha value of 0.971. The coefficients after deleting any single item ranged from 0.963 to 0.968, none of which were higher, also indicating high internal consistency. In summary, the Cronbach's alpha values for all latent variables were above 0.70, indicating that the measurement tools have good internal consistency and the reliability level meets the requirements of empirical research.

**Table 6:** Cronbach's alpha reliability values for latent variables

Variable Name	Item Code	Cronbach's Alpha if Item Deleted	Cronbach's Alpha
General Self-Efficacy	GSES1	0.936	0.936
	GSES2	0.936	
	GSES3	0.932	
	GSES4	0.927	
	GSES5	0.926	
	GSES6	0.930	
	GSES7	0.927	
	GSES8	0.927	
	GSES9	0.928	
	GSES10	0.925	
Comprehensive Literacy Perceived Improvement	CL-PIS1	0.968	0.971
	CL-PIS2	0.965	
	CL-PIS3	0.963	
	CL-PIS4	0.963	
	CL-PIS5	0.964	
	CL-PIS6	0.965	

Validity analysis

Exploratory Factor Analysis

The Comprehensive Literacy Perceived Improvement scale was an adapted scale, requiring exploratory factor analysis. Before conducting EFA, the suitability of its data was tested, with results shown in Table 7. The Kaiser-Meyer-Olkin (KMO) measure of sampling adequacy was 0.940, reaching the excellent level above 0.80, indicating good commonality among variables and suitability for factor analysis. Meanwhile, Bartlett's test of sphericity had an approximate chi-square value of 2050.898 with 15 degrees of freedom and a significance level of Sig. = 0.000 (p < 0.001), indicating that the correlation matrix is not an identity matrix and there are significant correlations among the variables, satisfying the basic conditions for factor analysis. Therefore, further factor extraction and structure verification could be performed for the comprehensive literacy perceived improvement variable.

**Table 7:** KMO and Bartlett's test for comprehensive literacy perceived improvement KMO: Kaiser-Meyer-Olkin

KMO Measure of Sampling Adequacy		.940
Bartlett's Test of Sphericity	Approx. Chi-Square	2050.898
	df.	15
	Sig.	0.000

After performing principal component analysis on the Comprehensive Literacy Perceived Improvement scale, one common factor with an eigenvalue greater than one was extracted. This factor could explain 87.18% of the total variance, indicating a highly concentrated scale structure with a good single-factor structure. The factor loadings of all items were high, ranging from 0.910 to 0.945, indicating strong overall consistency and good representativeness of the items. In summary, the Comprehensive Literacy Perceived Improvement scale has a clear single-factor structure and high explanatory power, making it suitable for subsequent confirmatory factor analysis and structural model construction (Table 8).

**Table 8:** Component matrix and variance explanation rate for the comprehensive literacy perceived improvement scale

Dimension	Item Code	Factor	Component	Variance Explained
Comprehensive Literacy Perceived Improvement	CL-PIS1	1	0.948	87.18%
	CL-PIS2	1	0.945	
	CL-PIS3	1	0.938	
	CL-PIS4	1	0.931	
	CL-PIS5	1	0.929	
	CL-PIS6	1	0.910	
Cumulative Variance Contribution Rate				87.18%

Confirmatory Factor Analysis

Model fit assessment: This study used AMOS 29.0 software to perform confirmatory factor analysis on the latent variable "Comprehensive Literacy Perceived Improvement" and its corresponding items. First, the goodness-of-fit of the measurement model was tested. Various fit indices are shown in Table 9, all of which reached ideal standards, indicating that the measurement model fits the actual data well, allowing for subsequent validity analysis.

**Table 9:** Measurement model fit indices X/DF: Chi-Square / Degrees of Freedom Ratio, RMSEA: Root Mean Square Error of Approximation, NFI: Normed Fit Index, IFI: Incremental Fit Index, CFI: Comparative Fit Index, GFI: Goodness of Fit Index, AGFI: Adjusted Goodness of Fit Index

Fit Index	X/DF	RMSEA	NFI	IFI	CFI	GFI	AGFI
Evaluation Criterion	1-3	<0.08	>0.9	>0.9	>0.9	>0.9	>0.9
Model Index	2.386	0.073	0.990	0.994	0.994	0.973	0.938
Result Judgment	Excellent	Excellent	Excellent	Excellent	Excellent	Excellent	Excellent

Convergent validity was assessed using three indicators: factor loadings, composite reliability (CR), and average variance extracted (AVE). As shown in Table 10, the standardized factor loadings of all observed variables on their measured latent variable were greater than 0.6 and significant at the p < 0.001 level. The CR value for comprehensive literacy perceived improvement was 0.971, higher than the recommended value of 0.7; the AVE value was 0.846, higher than the recommended standard of 0.5. This indicates that the measurement items for each latent variable have high consistency and good convergent validity.

**Table 10:** Convergent validity analysis for comprehensive literacy perceived improvement *p < 0.05, **p < 0.01, ***p < 0.001

Latent Variable	Observed Item	Std. Estimate	S.E.	T-value	P	SMC	CR	AVE
Comprehensive Literacy Perceived Improvement	CL-PIS1	0.883	0.070	9.113	***	0.780	0.971	0.846
	CL-PIS2	0.915	0.017	10.291	***	0.837		
	CL-PIS3	0.936	0.013	9.755	***	0.876		
	CL-PIS4	0.942	0.011	9.098	***	0.887		
	CL-PIS5	0.929	0.010	8.798	***	0.863		
	CL-PIS6	0.913	0.011	9.374	***	0.834		

Structural equation model hypothesis testing

Model Fit Assessment and Path Relationship Testing

After verifying the reliability and validity of the measurement model, this study used AMOS 29.0 software to test the structural model to verify the proposed research hypotheses. The fit of the structural model was analyzed first, with the following results: χ²/df = 2.839, RMSEA = 0.084, NFI = 0.922, CFI = 0.948, IFI = 0.948, GFI = 0.869, AGFI = 0.829. The model fit test results showed that the χ²/df value was 2.839, less than the lenient standard of 3 [23]; the RMSEA value was 0.084, slightly higher than the ideal standard of 0.08 [24], but still within an acceptable range [25-26]; and the relative fit indices NFI, CFI, and IFI were all above the good standard of 0.90, with CFI and IFI closer to the excellent level of 0.95 [24]. Although the absolute fit indices GFI and AGFI were slightly below 0.90, it is generally accepted in the academic field that these two indices are susceptible to sample size and model complexity. In social science research, when their values are greater than 0.80 or 0.85, combined with other good fit indices, the model can still be accepted [23]. Judging comprehensively by various indices, the overall fit of this structural equation model with the empirical data ranges from acceptable to good, and it can be used for subsequent path coefficient and hypothesis testing.

Parameter estimation was then performed using the maximum likelihood method. The test results for each path coefficient are shown in Table 11.

**Table 11:** Structural Equation Model Path Relationship Test Results (N=264) *p < 0.05, **p < 0.01, ***p < 0.001

Path Relationship	Std. Estimate	S.E.	C.R.	P	Label
General Self-Efficacy ⟵ Extracurricular Part. Freq.	0.137	0.02	2.147	*	a
Comp. Literacy Perc. Imp. ⟵ General Self-Efficacy	0.532	0.158	7.456	***	c
Comp. Literacy Perc. Imp. ⟵ Extracurricular Part. Freq.	0.197	0.036	3.768	***	b

Based on the analysis results in Table 11, the hypotheses proposed in this study were tested one by one:

Hypothesis H1 proposed that "Extracurricular activity participation frequency has a significant positive impact on general self-efficacy." Path analysis showed that the path coefficient from extracurricular activity participation frequency to general self-efficacy was 0.137, with a critical ratio of 2.147, reaching significance level (p < 0.05). Therefore, hypothesis H1 is supported.

Hypothesis H2 proposed that "General self-efficacy has a significant positive impact on comprehensive literacy perceived improvement." Path analysis showed that the path coefficient from general self-efficacy to comprehensive literacy perceived improvement was 0.532, with a critical ratio of 7.456, highly significant at the p < 0.001 level. Therefore, hypothesis H2 is supported.

Hypothesis H3 proposed that "Extracurricular activity participation frequency has a significant positive impact on comprehensive literacy perceived improvement." Path analysis showed that the direct path coefficient from extracurricular activity participation frequency to comprehensive literacy perceived improvement was 0.197, with a critical ratio of 3.768, highly significant at the p < 0.001 level. Therefore, hypothesis H3 is supported.

Mediation Effect Test

To test whether general self-efficacy plays a mediating role between extracurricular activity participation frequency and comprehensive literacy perceived improvement, this study used the bootstrap sampling method for mediation effect testing. In AMOS, the number of bootstrap samples was set to 5000, and the 95% confidence interval for the effect value was calculated. The mediation effect analysis results are shown in Table 12. The estimated value of the mediation effect was 0.073, and its 95% bootstrap confidence interval was (0.004, 0.106). Since this interval does not include 0, it indicates that the mediating effect of general self-efficacy is significant. Within the total effect, the proportion of the direct effect was 72.96%, while the proportion of the indirect effect generated through general self-efficacy was 27.04%.

**Table 12:** Bootstrap mediation effect test results

Effect Relation	Effect Value	LLCI	ULCI	Effect Proportion	Conclusion
Total Effect	0.270	0.161	0.380	100%	Significant
Direct Effect	0.197	0.098	0.308	72.96%	Significant
Indirect Effect	0.073	0.004	0.106	27.04%	Significant

In summary, general self-efficacy plays a partial mediating role between extracurricular activity participation frequency and comprehensive literacy perceived improvement. This means that extracurricular activity participation frequency can not only directly positively affect students' perceived improvement in comprehensive literacy but can also indirectly promote the improvement of their comprehensive literacy by enhancing their general self-efficacy.

## Discussion

This study systematically examined the mechanism through which the frequency of extracurricular activity participation influences the perceived improvement of comprehensive literacy among medical students and verified the mediating role of general self-efficacy, using structural equation modeling and the Bootstrap method. The main findings supported all research hypotheses. These results are discussed below.

Summary of main findings

This study found that extracurricular activity participation can not only directly and positively predict the perceived improvement of comprehensive literacy among medical students but also exert an indirect facilitating effect by enhancing their general self-efficacy. General self-efficacy played a significant partial mediating role between the two. This finding clearly reveals the intrinsic psychological mechanism through which extracurricular activities influence student development, namely, the pathway of "environmental stimulus (extracurricular activities) → individual cognition (self-efficacy) → developmental outcome (comprehensive literacy)."

The direct effect of extracurricular activity participation frequency on perceived improvement of comprehensive literacy

The results confirmed that extracurricular activity participation has a significant positive direct effect on the perceived improvement of comprehensive literacy (H3 supported). This finding aligns with numerous previous studies, indicating that extracurricular activities provide students with authentic contexts to apply classroom knowledge, exercise practical problem-solving skills, and engage in teamwork and communication. Particularly for medical students, extracurricular activities such as research projects, social practice, and volunteer services are crucial arenas for cultivating their innovation, collaboration, communication, and critical thinking skills. The "dose-response" relationship revealed by the one-way ANOVA, where higher participation frequency was associated with stronger perceived literacy improvement, further strengthens this conclusion. It suggests that sustained and in-depth participation yields greater benefits than superficial or occasional involvement.

The mediating role of general self-efficacy

This study further revealed the key mediating role of general self-efficacy (H4 supported). The indirect effect of extracurricular activity participation on the perceived improvement of comprehensive literacy through general self-efficacy was significant, accounting for 27.04% of the total effect. This result corroborates Bandura's social cognitive theory. Extracurricular activities often involve challenging tasks. When students participate in these activities and successfully overcome challenges, they gain valuable "mastery experiences," which are the primary source of high self-efficacy. Furthermore, observational learning from peers, encouragement from instructors, and positive emotional arousal within the activities collectively constitute multiple pathways for enhancing self-efficacy. Once students develop a firm belief in "I can do it," this belief transfers to broader learning and developmental contexts. Individuals with high self-efficacy set more challenging goals, exert greater effort, and demonstrate stronger resilience in the face of setbacks [27]. These positive cognitive and behavioral patterns ultimately contribute significantly to their improvement in comprehensive literacy.

Practical implications

This study applies social cognitive theory to the domain of extracurricular activities and development among medical students. Empirically testing a specific mediation model provides a clear explanation of the psychological mechanism underlying "how extracurricular activities work," addressing the previous focus on simple correlations in much of the literature.

Regarding practical implications, this study offers clear guidance for educational administrators in medical schools:

Encourage and Create Participation Opportunities

Schools should attach great importance to the educational value of extracurricular activities, systematically design the activity system to ensure alignment with comprehensive literacy cultivation goals, and encourage students, especially those with low participation, to engage actively.

Focus on Fostering Self-Efficacy Within Activities

Activity design should not only focus on the form and outcome but also pay attention to the cultivation of psychological capital during the process. Instructors should consciously set challenges for students that are "within reach with effort," provide timely constructive feedback and encouragement, and create opportunities for students to experience success, thereby specifically enhancing their general self-efficacy.

Limitations and future research directions

This study also has several limitations. First, the use of cross-sectional survey data makes it difficult to establish strict causal relationships between variables, although the mediation effect was verified statistically. Future research could employ longitudinal designs or experimental interventions to further validate the causal chain. Second, the sample was drawn from a single medical school, which may limit the representativeness and generalizability of the findings. Future multi-center studies with larger samples are needed to enhance the external validity of the conclusions. Finally, the measurement of comprehensive literacy relied on student self-reports. Future studies could incorporate objective indicators such as teacher evaluations and behavioral observations to provide a more comprehensive measurement. Furthermore, while the current model accounted for key variables, it may not have fully captured the potential influence of unmeasured confounding factors (e.g., participants' prior academic performance, personality traits, or socioeconomic status). Future research should incorporate more comprehensive control variables and utilize advanced statistical methods to better address these potential confounding effects.

## Conclusions

Based on empirical analysis, this study draws the following main conclusions: a significant positive relationship exists between the frequency of extracurricular activity participation and the level of perceived comprehensive literacy improvement among medical students. This relationship exhibits a "dose-response" pattern, meaning more frequent participation is associated with greater perceived gains. Extracurricular activity participation not only directly contributes to the enhancement of medical students' comprehensive literacy but also fosters it indirectly by boosting their general self-efficacy.

General self-efficacy plays a significant partial mediating role between participation frequency and perceived comprehensive literacy improvement. This indicates that extracurricular activities promote development both directly and indirectly through the crucial pathway of "empowerment”, strengthening students' psychological belief in their capabilities. In summary, extracurricular activities constitute an indispensable component in cultivating outstanding medical talents. While emphasizing classroom instruction, higher education institutions should focus on building a supportive extracurricular activity system. Furthermore, the cultivation of self-efficacy should be skillfully integrated into the design and guidance of these activities. This dual approach, leveraging both direct involvement and indirect empowerment, will more effectively promote the holistic development of medical students' comprehensive literacy.

## Appendices

English translation of the questionnaire

**Table 13:** Demographic profile of medical student participants

Part I - Demographic characteristics	
1. Your gender:	○ Male ○ Female
2. Your grade:	○ Year 1 ○ Year 2 ○ Year 3 ○ Year 4 ○ Year 5
3. During the past semester, how frequently did you participate in extracurricular activities?	○ Never participated ○ Participated 1-3 times in total ○ Participated 4-6 times in total ○ Participated 7-9 times in total ○ Participated 10 times or more in total

**Table 14:** General self-efficacy scale In accordance with the scale's usage permissions, the full scale is not reproduced in the appendix. Only one sample item is provided.

Part II -Sample Item from the General Self-Efficacy Scale	
4. If I try hard enough, I can always manage to solve difficult problems.	○Strongly Disagree ○ Disagree ○ Agree ○Strongly Agree

**Table 15:** Comprehensive literacy perceived improvement scale

Part III - Comprehensive Literacy Perceived Improvement	
15. Through participating in extracurricular activities, I have become better at viewing and reflecting on a problem from multiple perspectives.	○Strongly Disagree ○Disagree ○Neutra ○Agree ○Strongly Agree
16. Through participating in extracurricular activities, I can generate more creative ideas and apply them to practical tasks.	○Strongly Disagree ○Disagree ○Neutra ○Agree ○Strongly Agree
17. Through participating in extracurricular activities, I have become more adept at collaborating and organizing tasks within a team.	○Strongly Disagree ○Disagree ○Neutra ○Agree ○Strongly Agree
18. Through participating in extracurricular activities, I take initiative and responsibility more readily.	○Strongly Disagree ○Disagree ○Neutra ○Agree ○Strongly Agree
19. Through participating in extracurricular activities, I pay more attention to social issues and am more willing to participate in public welfare or volunteer services.	○Strongly Disagree ○Disagree ○Neutra ○Agree ○Strongly Agree
20. Through participating in extracurricular activities, I believe in my ability to overcome challenges and achieve my goals.	○Strongly Disagree ○Disagree ○Neutra ○Agree ○Strongly Agree

The original questionnaire (in Chinese)

**Table 16:** Demographic profile of medical student participants (original Chinese version)

Part I - 人口学特征	
1.您的性别：	○男 ○女
2.您的年级	○大学一年级 ○大学二年级 ○大学三年级 ○大学四年级 ○大学五年级
3.过去的一学期，您参与第二课堂活动的频率是：	○一次都没参加 ○整个学期参加：1-3次 ○整个学期参加：4-6次 ○整个学期参加：7-9次 ○整个学期参加10次及以上

**Table 17:** General self-efficacy scale (original Chinese version) In accordance with the scale's usage permissions, the full scale is not reproduced in the appendix. Only one sample item is provided.

PartII -一般自我效能感示例题目	
4.如果我尽力去做的话，我总是能够解决问题的。	○非常不符合 ○ 不符合 ○ 符合 ○非常符合

**Table 18:** Comprehensive literacy perceived improvement scale (original Chinese version)

Part III - 综合素养感知提升	
15.通过参加通过参加第二课堂活动，我更善于从多个角度看待和反思一个问题。	○十分不同意 ○ 不同意 ○ 不一定 ○ 同意 ○十分同意
16.通过参加第二课堂活动，我能提出更有创意的想法，并应用于实践任务中。	○十分不同意 ○ 不同意 ○ 不一定 ○ 同意 ○十分同意
17.通过参加第二课堂活动，我在团队合作中更擅长协作与组织任务。	○十分不同意 ○ 不同意 ○ 不一定 ○ 同意 ○十分同意
18.通过参加第二课堂活动，我能更主动承担责任。	○十分不同意 ○ 不同意 ○ 不一定 ○ 同意 ○十分同意
19.通过参加第二课堂活动，我更关注社会问题，并愿意参与公益或志愿服务。	○十分不同意 ○ 不同意 ○ 不一定 ○ 同意 ○十分同意
20.通过参加第二课堂活动，我相信自己能够克服挑战并实现目标。	○十分不同意 ○ 不同意 ○ 不一定 ○ 同意 ○十分同意
